# Studies of royal jelly and associated cross-reactive allergens in atopic dermatitis patients

**DOI:** 10.1371/journal.pone.0233707

**Published:** 2020-06-02

**Authors:** Taketoshi Hata, Takako Furusawa-Horie, Yasuko Arai, Tomoko Takahashi, Mariko Seishima, Kenji Ichihara

**Affiliations:** 1 Nagaragawa Research Center, API Co., Ltd., Gifu, Japan; 2 Department of Dermatology, Gifu University Graduate School of Medicine, Gifu, Japan; University of Maryland School of Medicine, UNITED STATES

## Abstract

Royal jelly (RJ), a creamy substance secreted by honeybees, is the exclusive diet for queen bee differentiation and life maintenance. RJ has been used in cosmetics, beverages, medicines, and supplements worldwide. However, allergy is a concerning issue for RJ, especially in atopic dermatitis (AD) and asthma patients. In some cases, allergic reactions are seen after the first intake of RJ, suggesting the existence of allergens cross-reactive with RJ. Information about the cross-reactive allergens is very important for the safe application of RJ; however, study of this cross-reactivity is quite limited. In this study, we attempted to identify allergens cross-reactive with RJ by using serum samples from 30 AD patients who had never been exposed to RJ. In an enzyme-linked immunosorbent assay (ELISA) experiment, RJ-binding IgE antibodies were detected in the serum of 10 out of 30 patients, and their antibody titers ranged from 4- to 2,048-fold dilution ratios. Additionally, 3 AD patients were determined to be positive in a skin-prick test (SPT) with an RJ solution. Significant correlations were observed between the anti-RJ antibody titer and nonspecific IgE and between the anti-RJ antibody titer and the Eczema Area and Severity Index score. We further examined the cross-reactivity between RJ and 14 typical allergens by using an ELISA-inhibition assay and demonstrated that the following 6 allergens showed cross-reactivity with RJ: the European house dust mite (HDM) (*Dermatophagoides pteronyssinus*), American HDM (*Dermatophagoides farinae*), snow crab (*Chionocetes spp*.*)*, edible crab (*Cancer pagurus*), German cockroach (*Blatella germanica*), and honeybee venom (*Apis mellifera*). In conclusion, people with a history of allergic diseases, including AD, asthma, and allergic rhinitis, should be cautioned against consuming RJ products because of the potential for cross-reactive responses to ensure the safe and successful use of RJ supplements.

## Introduction

Royal jelly (RJ) is secreted from the hypopharyngeal and mandibular glands of worker honeybees, and it is the exclusive diet for queen bee differentiation and life maintenance [1–4]. Compared with other female worker bees, a queen honeybee that is fed RJ exclusively throughout her life has a larger body, a 20-fold longer lifetime, and well-developed gonads [5]. RJ is a creamy yellow-white, acidic, astringent material composed of water (62.0–68.5%), proteins (11–18%), sugars (7–18%), lipids (2–8%), amino acids, vitamins, and minerals [6]. Health-benefitting and pharmaceutical effects of RJ, such as the improvement of reproductive-system-associated issues [7], the skin barrier [8], and mental health [9], have been reported; therefore, RJ has been used in dietary supplements, medicines, and cosmetics worldwide.

On the other hand, adverse events induced by RJ have been reported in recent decades, almost all of which are allergy-associated symptoms. These symptoms range from mild to severe and include rhinitis, eczema, contact dermatitis, urticaria, conjunctivitis, hemorrhagic colitis, acute asthma, bronchospasm, and fatal anaphylaxis [10–21]. Allergy sufferers such as asthma and atopic dermatitis (AD) patients, probably have a risk of allergic reaction to RJ. It was reported that 52% of allergic patients (n = 75) in Australia had a positive immunoglobulin E (IgE) response to RJ [14]. Leung et al. also showed that, in Hong Kong, 16.8% of 666 adult asthma patient serum samples contained RJ-reactive IgE [22]. These patients may be sensitized by RJ products; however, it is also suggested that there are unknown allergens cross-reactive with RJ.

The existence of allergens cross-reactive with RJ has been suggested for a long time because allergic symptoms have been observed following the first intake of RJ in several cases [13, 18, 23–30]. Few short reports on the cross-reactivity of RJ are available. Vila et al. reported a case of anaphylaxis following the first intake of RJ and demonstrated the cross-reactivity between RJ and the European house dust mite (HDM) *Dermatophagoides pteronyssinus* by immunoblot-inhibition assay analysis of the patient’s serum [24]. Li et al. showed cross-reactivity between RJ and honeybee venom in a patient with occupational allergy who had been sensitized by both RJ and honeybee venom [31].

However, the causality underlying RJ allergic reaction following the first instance of ingestion is not well understood, even though serious allergic reactions have occurred in many cases. Therefore, the discovery of missing information about allergens cross-reactive with RJ is crucial to avoid future unnecessary accidents. In the present study, we aimed to assess whether allergens cross-reactive with RJ exist in AD patients.

## Materials and methods

### Subjects

The study was approved by the Gifu University Hospital Independent Ethics Committee (29–090, 30–034), and written, informed patient consent was obtained. Serum samples were obtained from 30 outpatients with AD who visited the Department of Dermatology, Gifu University Hospital. Eligibility criteria were age 20 or older and a diagnosis of AD. We confirmed that all patients had no previous contact with RJ in their interviews. All 30 patients met the inclusion criteria and were recruited for the study. Thermo Scientific^TM^ ViewAllergy^TM^39 (multiple-allergen simultaneous tests using semiquantitative ImmunoCAP^TM^, Thermo Fisher Diagnostics K.K., Tokyo, Japan), nonspecific IgE titers, Eczema Area and Severity Index (EASI) scores, thymus and activation-regulated chemokine (TARC) levels, eosinophil counts, lactate dehydrogenase (LD) levels, levels of ImmunoCAP^®^ Specific IgE against the American HDM (*Dermatophagoides farinae*) and honeybee venom (*Apis mellifera*), and levels of IgE antibodies specific for HDM allergen components were measured. Clinical information and experimental data from the AD patients are shown in Table 1. Serum samples from RJ-exposed factory workers, who had allergies to RJ but not to other allergens, were also used for Western blotting analysis, and their clinical information is described in the supporting information (S1 Table).

**Table 1:** Clinical information and experimental data for the AD patients in the study.

Subject no.†	Age	Sex	Severity	Onset	TARC (pg/mL)	Eosino-phils (%)	Eosino-phils (cells/μL)	LD (124–222 U/L)	EASI	Nonspecific IgE (IU/mL)	RJ-specific antibody titer (fold)	Log_2_ RJ antibody titer	Allergy history
31	31	M	mild	childhood	268	2.4	133	ND	2.1	288	0	0	allergic rhinitis
32	41	F	severe	childhood	14439	31.7	2447	194	25.0	199	0	0	
33	32	F	severe	childhood	6167	18.6	1793	201	26.5	2050	0	0	
34	54	M	severe	childhood	1202	4.0	221	ND	33.5	12086	32	5	
35	22	M	moderate	childhood	1272	5.7	536	306	8.3	2454	0	0	allergic rhinitis
36	23	M	moderate	childhood	854	3.9	381	ND	19.9	9110	512	9	
37	21	M	severe	childhood	3329	32.5	2379	409	45.8	13110	128	7	
38	25	F	mild	juvenile	2030	12.5	1325	218	10.3	2663	8	3	allergic rhinitis
39	33	M	severe	childhood	4937	13.4	829	382	54.8	31946	2048	11	
40	35	F	moderate	childhood	539	2.6	287	ND	8.5	158	0	0	
41	36	M	moderate	childhood	759	7.0	859	233	9.2	675	0	0	
42	63	F	moderate	childhood	770	4.4	272	196	16.3	2411	4	2	
43	47	F	mild	childhood	260	5.3	359	ND	3.2	1210	0	0	allergic rhinitis
44	32	M	severe	juvenile	4419	9.4	619	319	48.3	52624	1024	10	
45	55	F	mild	adult	1016	2.6	217	ND	5.5	1002	0	0	
46	26	F	moderate	childhood	266	3.9	190	125	10.9	490	0	0	
47	47	M	mild	adult	282	0.0	0	234	2.8	32	0	0	
48	26	F	severe	childhood	1002	15.0	1067	195	40.2	3469	64	6	allergic rhinitis
49	27	M	moderate	childhood	1180	N.D.	N.D.	222	7.7	436	0	0	allergic rhinitis
50	24	M	severe	childhood	3565	8.0	758	422	18.5	6577	0	0	
51	24	M	moderate	childhood	1932	8.9	473	ND	21.3	6822	16	4	
52	36	F	moderate	childhood	4758	9.0	883	143	13.5	152	0	0	
53	23	M	mild	childhood	451	7.3	418	ND	3.3	679	0	0	
54	28	M	severe	childhood	26845	26.0	2192	206	30.3	19521	0	0	
55	42	M	severe	juvenile	4752	10.5	549	253	20.2	1928	0	0	
56	38	M	moderate	adult	11151	7.0	924	402	15.6	1303	0	0	
57	35	F	mild	childhood	333	5.1	229	150	4.3	576	0	0	
58	49	F	mild	childhood	525	3.0	72	ND	9.6	1161	8	3	
59	20	F	moderate	adult	938	9	498	ND	10.8	2688	0	0	
60	22	F	mild	adult	458	2.5	208	176	5.4	83	0	0	

† These subjects had never been exposed to royal jelly according to their self-reports.

### Allergen sample preparation

Extracts of crude bodies of the European HDM (*Dermatophagoides pteronyssinus*) and the American HDM (*Dermatophagoides farinae*) were purchased from Biostir Inc. (Osaka, Japan). Custom-made allergen extracts of cat dander (*Felis domesticus*), timothy grass pollen (*Phleum pretense*), Japanese cedar pollen (*Cryptomeria japonica*), and Japanese mugwort pollen (*Artemisia indica*) were purchased from the Institute of Tokyo Environmental Allergy Inc. (Tokyo, Japan). They were prepared at a protein concentration of 3.0 mg/mL with 0.01 M Phosphate-buffered saline (PBS) by Lowry’s method. We adjusted the protein concentration to 160 μg/mL with 0.01 M PBS before use. Lyophilized raw materials from buckwheat (*Fagopyrum esculentum*) and peanut (*Arachis hypogaea*) were purchased from Stallergenes Greer plc (London, United Kingdom). The Alaskan pink shrimp (*Pandalus borealis*), German cockroach *(Blatella germanica*), silk moth (*Bombyx mori*), and edible crab (*Cancer pagurus*) were purchased from Biostar Inc., and we obtained honeybee venom (*Apis mellifera*) from LATOXAN (Portes lès Valence, France). Freshly frozen snow crab (*Chionocetes spp*.) was purchased from Kamasho (Fukushima, Japan), and both outer shells and leg muscles were lyophilized and powdered. The lyophilized powders were dissolved in 0.01 M PBS (pH 7.4) at a 1:30 weight-to-volume ratio and stirred overnight at 4ºC. After extraction and centrifugation (15,000 ×g for 30 minutes), the supernatants were filtered with the 0.45-μm membrane DISMIC^®^-13HP (ADVANTECH, Tokyo, Japan), and their protein concentrations were measured by using the Pierce™ BCA Protein Assay Kit (Thermo Fisher Scientific, Waltham, MA) and adjusted to 160 μg/mL with 0.01 M PBS. Lyophilized RJ was obtained from API Co., Ltd. (Gifu, Japan).

### ELISA

IgE recognition of RJ proteins by AD patient serum samples and their antibody titers were evaluated by ELISA. ELISA plates (Nunc MaxiSorp™ flat bottom, Thermo Fisher Scientific) were coated with 7.5 μg/mL lyophilized RJ in 0.05 M carbonate/bicarbonate buffer (pH 9.6) and incubated for 1 h at 37ºC. Blocking was performed with EzBlockChemi (ATTO, Tokyo, Japan) for 1 h at 37ºC. Serially diluted serum samples were added to the ELISA plates and incubated overnight at 4ºC. RJ-binding IgE antibodies were detected by incubation with a horseradish peroxidase (HRP)-conjugated mouse anti-human IgE Fc antibody (Abcam, Cambridge, United Kingdom) diluted 16,000-fold in Tris-buffered saline with Tween^®^20 (TBS-T tablets, pH 7.6; Takara Bio Inc., Shiga, Japan) for 1 h at 37ºC, followed by incubation with a TMB substrate (SeraCare Life Sciences, Inc., Milford, MA) for 1 h at 37ºC. The reaction was stopped by adding 1 M phosphate, and the absorbance at 450 nm was measured. The plates were rinsed after each incubation step with TBS-T. All samples were assayed in triplicate. Commercial pooled human serum (Cosmo Bio Co., Ltd., Tokyo, Japan) was used as a negative control, and the TBS-T solution was used as the blank. The titers of antibodies specific for RJ are expressed as the highest dilution ratio in which a positive reaction was observed. A positive reaction, i.e., a positive IgE level, was defined as an absorbance greater than the mean value for the blanks plus 0.01.

### ELISA-inhibition assay

To study the cross-reactivity between RJ and other common allergens, an ELISA-inhibition assay was performed. The experimental procedure was the same as that in the ELISA experiment described above, except for preincubation with allergens. Diluted serum samples were preincubated overnight at 4ºC with an equal volume of an allergen extract solution or a TBS-T solution, which was used as a control. The final protein concentration of the allergen extracts was 80 μg/mL, and the dilution ratios of the serum samples were defined so that the absorbance of the control became approximately 0.10; thus, the dilution ratio of subject no. 39 was 256-fold, that of subject no. 44 was 64-fold, that of subject no. 36 and subject no. 37 was 32-fold, and that of subject no. 48 was 4-fold.

### Western blotting

Sodium dodecyl sulfate-polyacrylamide gel electrophoresis (SDS-PAGE) was carried out using a precast 10–20% gradient gel and the Real gel plate SDG-592 (BIO CRAFT, Tokyo, Japan). Lyophilized RJ was prepared at a concentration of 2 mg/mL in a sample buffer (62.5 mM Tris, 2 w/w% SDS, 10 w/v% glycerol, and 0.1 M dithiothreitol) and heated at 100ºC for 5 minutes, and then 143 μL of the solution was loaded into one large well (286 μg of RJ/143 μL). Precision Plus Protein™ Prestained Standards Dual Color (Bio-Rad, Hercules, CA) was used as a molecular weight marker. The gel-separated proteins were detected by Coomassie blue staining with EzStain Aqua (ATTO, Tokyo, Japan). Following SDS-PAGE, the gel-separated proteins were electrotransferred to polyvinylidene difluoride membranes (Clear Blot Membrane-P plus, ATTO), which were incubated with EzBlock Chemi at room temperature for 1 h, cut into strips (75 x 8 mm) and incubated overnight in 1 mL of AD patient serum at 4°C. After washing with TBS-T, the membranes were incubated at room temperature for 1 h with an HRP-conjugated mouse anti-human IgE Fc antibody (Abcam) diluted 10,000-fold in a TBS-T solution. The secondary antibody was detected with ECL Prime Western Blotting Detection Reagent (GE Healthcare Life Sciences, Marlborough, MA), and chemiluminescence was observed with Ez-Capture MG (ATTO).

### SPT

An SPT was performed by using undiluted, 1/10, and 1/100 raw RJ solutions for subject no. 38, subject no. 44, and subject no. 48, who showed a positive response to RJ by ELISA. Histamine dihydrochloride at a concentration of 10 mg/ml and a 0.9% saline solution were used as positive and negative controls, respectively. Skin reactions were estimated at 15 minutes after application. The longest wheal diameter (D) and the diameter perpendicular to D (d) were measured. The results are expressed as the mean calculated as (D + d)/2, with a result of 3 mm or greater being considered positive [32].

### Statistical analysis

Spearman’s rank correlation test was used to investigate the relationships between the anti-RJ antibody titer and the following assessment results for AD: the nonspecific IgE titer, EASI score, classification score based on the IgE of ViewAllergy^TM^39, TARC level, and LD level. Welch’s t-test was performed to compare ELISA-inhibition assays with and without preincubation with the test allergens. P values < 0.05, 0.01, and 0.001 were considered statistically significant. All analyses were performed using Ekuseru-Toukei 2012 ver.1.16 for Windows (Social Survey Research Information Co., Ltd., Tokyo, Japan).

## Results

### Detection of RJ-specific IgE antibodies in AD patients

**Fig 1:**
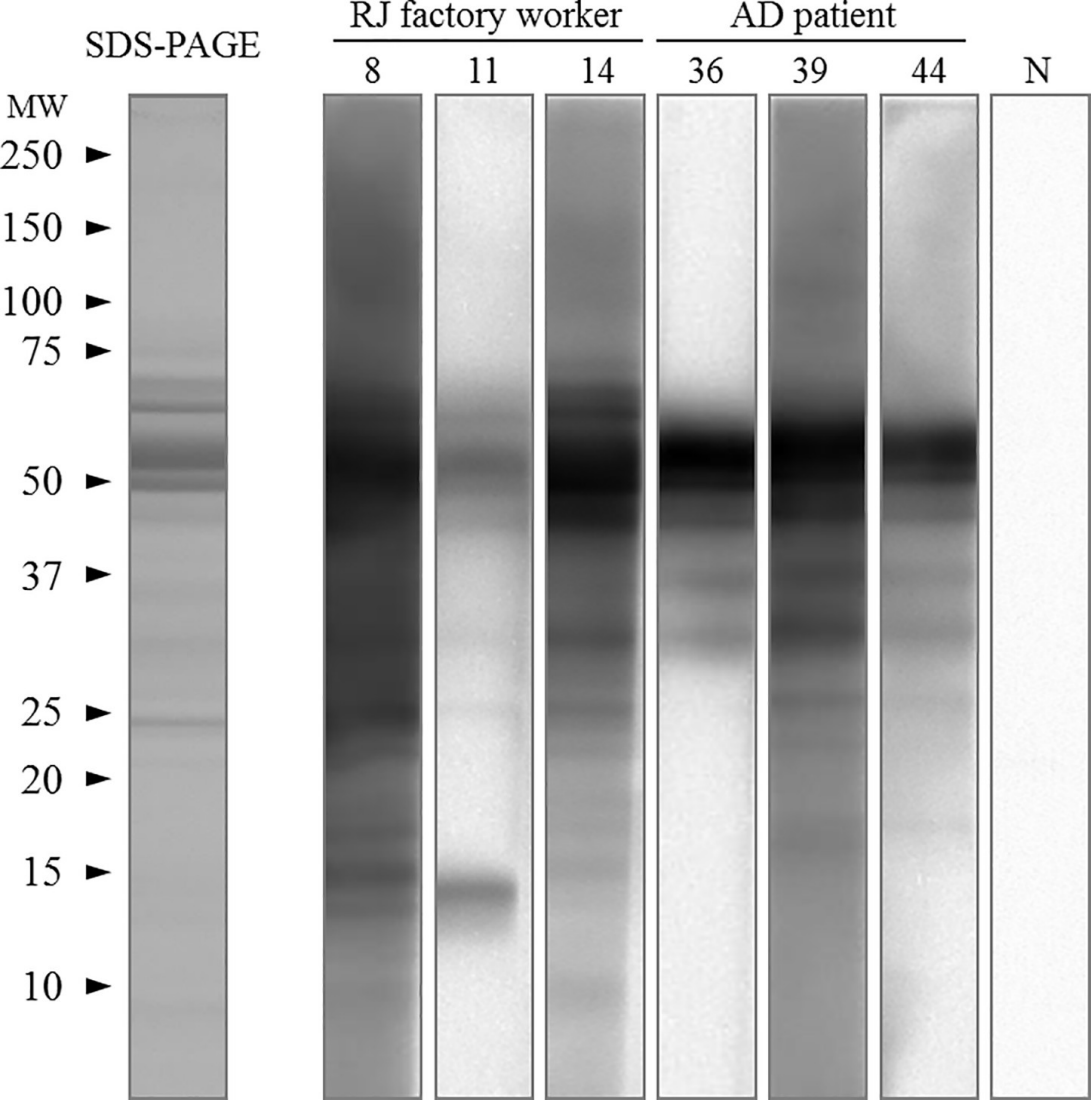
Western blot analysis of the RJ protein in serum samples from AD patients and RJ-exposed factory workers. RJ proteins were separated by 10–20% SDS-PAGE and stained with Coomassie brilliant blue (left lane). Immunoblot profiles were obtained for serum samples from AD patients (no. 36, no. 39, and no. 44) and occupational RJ-allergic patients (no. 8, no. 11, and no. 14). MW: molecular weight, and N: blank control.

Serum samples from AD patients were assessed by ELISA to detect RJ-specific IgE antibodies. In the ELISA experiment, 10 out of the 30 AD patients showed a positive result, and their antibody titers ranged from 4- to 2,048-fold (Table 1). Western blotting analysis was conducted using AD serum samples with relatively high antibody titers, specifically those from subject no. 36, subject no. 39, and subject no. 44. The IgE antibodies in the AD serum samples recognized similar proteins that were detected in serum samples from RJ-sensitized subjects no. 8, no. 11, and no. 14 (Fig 1). The results for nonspecific IgE antibodies and EASI scores are described in Table 1. The scores of multiple-allergen simultaneous tests, ViewAllergy^TM^39, are shown in S2 Table, and the results for TARC levels, eosinophils, and LD levels are shown in S3 Table.

### Correlations between the anti-RJ antibody titers and serum parameters

**Fig 2:**
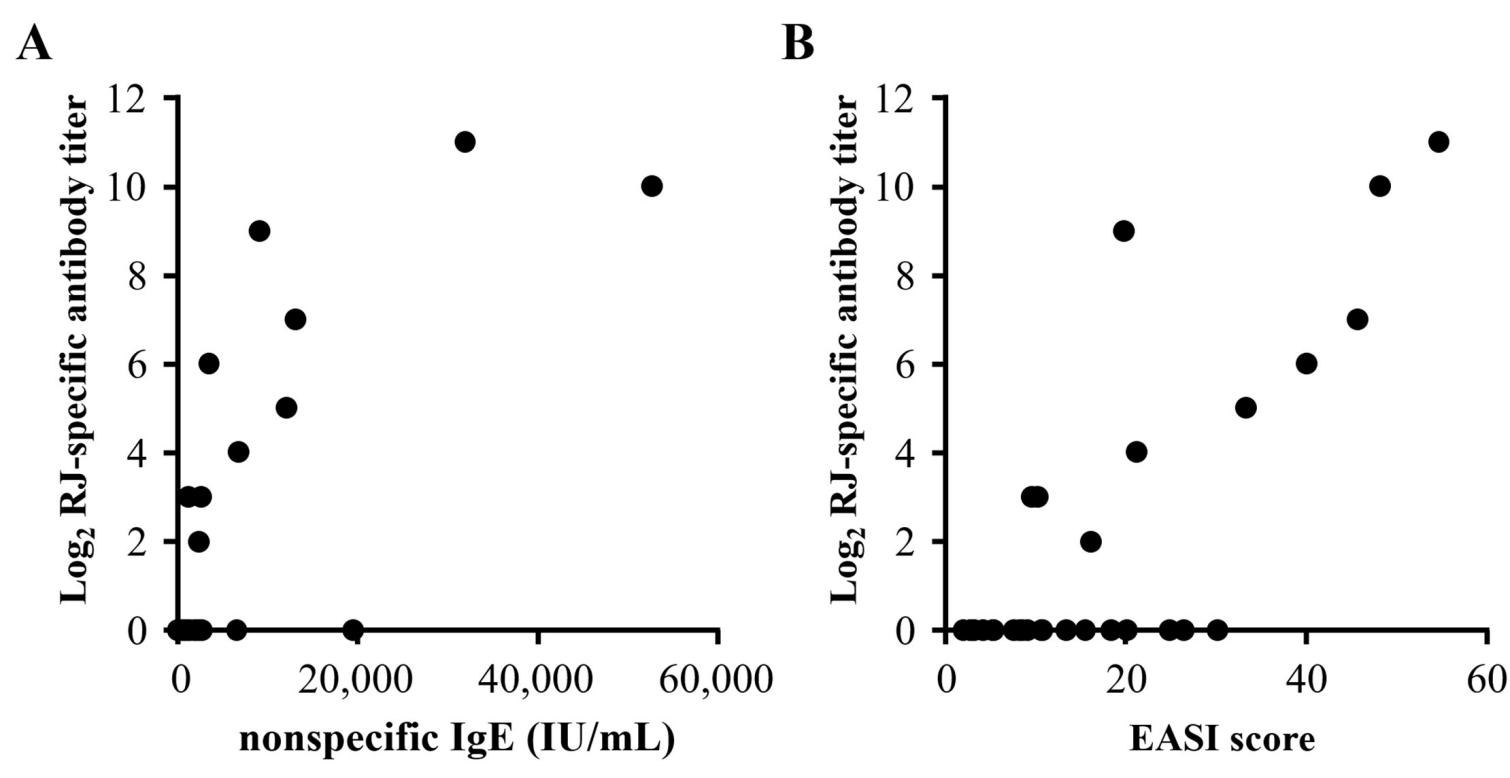
Correlations between anti-RJ antibody titers and clinical findings in the AD patients who participated in the study. The binary logarithm of the antibody titer was plotted, and negative results are represented as zero for convenience. Significant correlations were observed for nonspecific IgE (A) and the EASI score (B) by Spearman’s rank correlation test. rs represents Spearman’s rho (correlation coefficient).

Fig 2 shows the relationships between anti-RJ antibody titer and clinical examination findings. The anti-RJ antibody titer was significantly correlated with nonspecific IgE levels and EASI scores. Spearman’s rho values for nonspecific IgE levels or EASI scores against the antibody titer were 0.631 and 0.692, respectively (Fig 2A and 2B). TARC levels, eosinophils, and LD levels showed no significant correlation (S3 Table) with anti-RJ antibody titers. In addition, Table 2 shows the correlations between the anti-RJ antibody titer and ViewAllergy^TM^39 test scores. High correlation coefficients were observed for HDM (0.589), Malassezia (0.677), sesame seeds (0.669), shrimp (0.594), crab (0.670), buckwheat (0.597), banana (0.670), and cockroach (0.625).

**Table 2:** The correlation coefficients between anti-RJ antibody titers and the classification based on serum IgE levels in multiple-allergen simultaneous tests (View Allergy^TM^39).

Allergen	Spearman's rank correlation		
	*rs*	*p*	
Cocksfoot, Orchard grass	0.331	0.074	
Timothy grass	0.309	0.097	
Common ragweed	0.516	0.004	**
Mugwort	0.377	0.040	*
Japanese cedar	0.196	0.300	
Japanese cypress	0.298	0.109	
Grey alder	0.293	0.117	
Common silver birch	0.365	0.047	*
House dust mite	0.525	0.003	**
House dust 1	0.472	0.009	**
*Candida albicans*	0.546	0.002	**
*Alternaria alternata*	0.420	0.021	*
*Aspergillus fumigatus*	0.559	0.001	**
*Malassezia spp*.	0.681	< 0.001	***
Cat dander	0.519	0.003	**
Dog dander	0.374	0.042	*
Wheat	0.408	0.025	*
Soya bean, Soybean	0.489	0.006	**
Rice	0.433	0.017	*
Sesame seed	0.575	< 0.001	***
Tuna (Yellowfin tuna)	0.162	0.394	
Salmon (Atlantic)	0.379	0.039	*
Chub mackerel	0.083	0.664	
Shrimp	0.563	0.001	**
Crab	0.598	< 0.001	***
Cow's milk	-0.029	0.881	
Beef	0.422	0.020	*
Chicken	0.455	0.011	*
Pork	0.147	0.439	
Egg white	0.002	0.992	
Ovomucoid (egg white	0.187	0.323	
Buckwheat	0.528	0.003	**
Peanut	0.422	0.020	*
Apple	0.411	0.024	*
Kiwi	0.519	0.003	**
Banana	0.596	< 0.001	***
Latex	0.366	0.046	*
Cockroach (German)	0.589	< 0.001	***
Moth	0.398	0.030	*

### Identification of allergens cross-reactive with RJ

**Fig 3:**
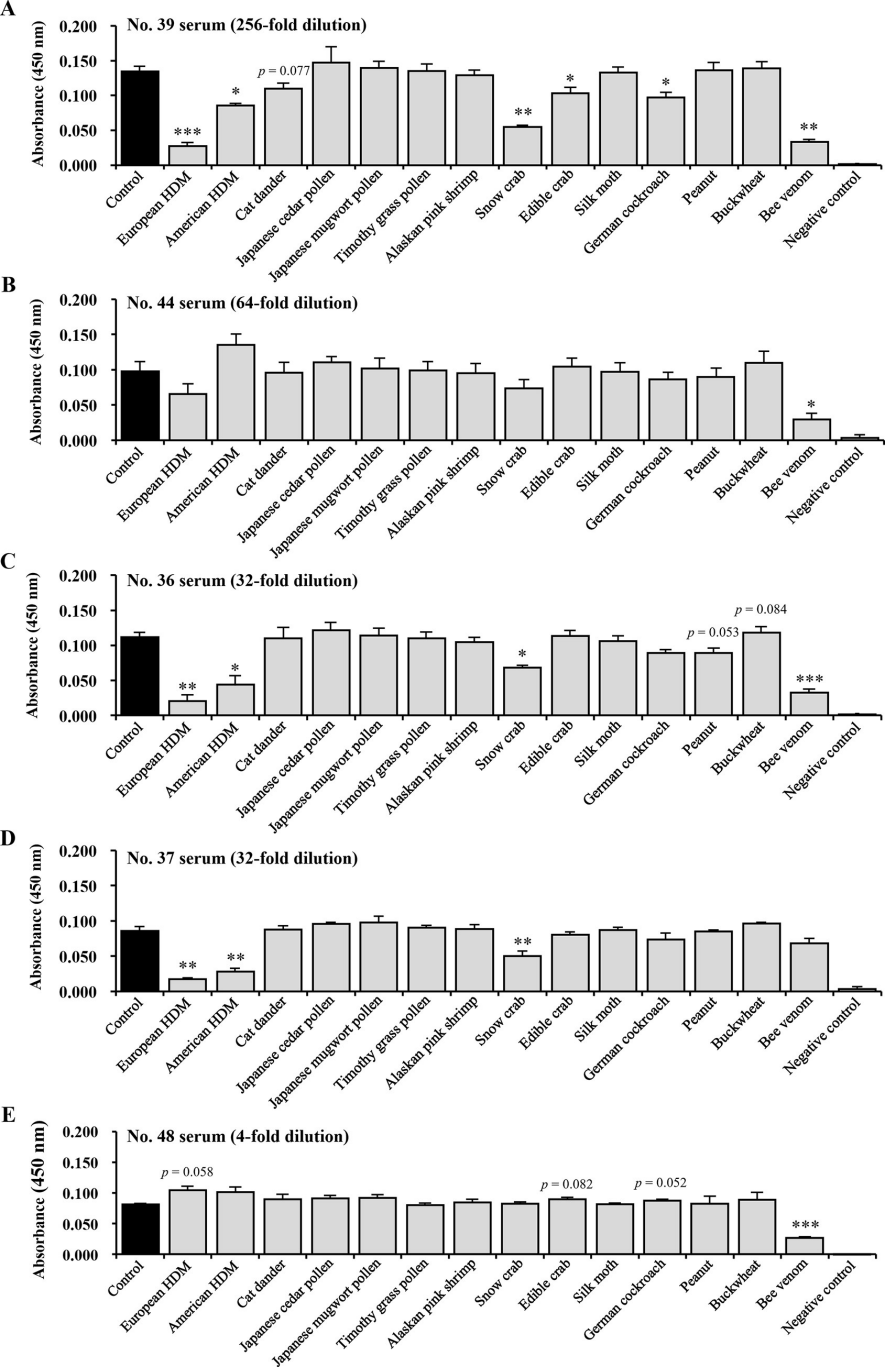
Cross-reactivity of RJ with major allergens demonstrated by an ELISA-inhibition assay with AD patient serum samples. Serum samples from subject no. 39 (A), no. 44 (B), no. 36 (C), no. 37 (D), and no. 48 (E) were used as representative samples in consideration of their relatively high anti-RJ antibody titers, and their serum dilution ratios are indicated in parentheses. The serum samples were preincubated with each allergen extract (80 μg/mL protein) or a TBS-T solution (control) prior to applying RJ in ELISA experiments. Pooled sera were used as a negative control. Data represent the mean ± the SEM of three independent experiments. Statistically significant differences were analyzed by Welch’s t-test: * p<0.05, ** p<0.01, and ***p<0.001 vs. the control. HDM is an abbreviation for house dust mite.

To investigate allergens cross-reactive with the RJ protein, 14 allergens were selected based on the results of the correlation test and their allergenic importance. Fig 3 shows the result of an ELISA-inhibition assay with 5 representative patient serum samples that were selected in consideration of their elevated anti-RJ antibody titers, as shown in Table 1. Significant inhibition of the response to RJ, shown as a decrease in absorbance, was observed with the European HDM (3/5), American HDM (3/5), snow crab (3/5), honeybee venom (4/5), edible crab (1/5), and German cockroach (1/5). However, cat dander, Japanese cedar pollen, Japanese mugwort pollen, timothy grass pollen, Alaskan pink shrimp, silkworm moth, peanut, and buckwheat did not significantly inhibit the response to RJ.

### SPT

**Fig 4:**
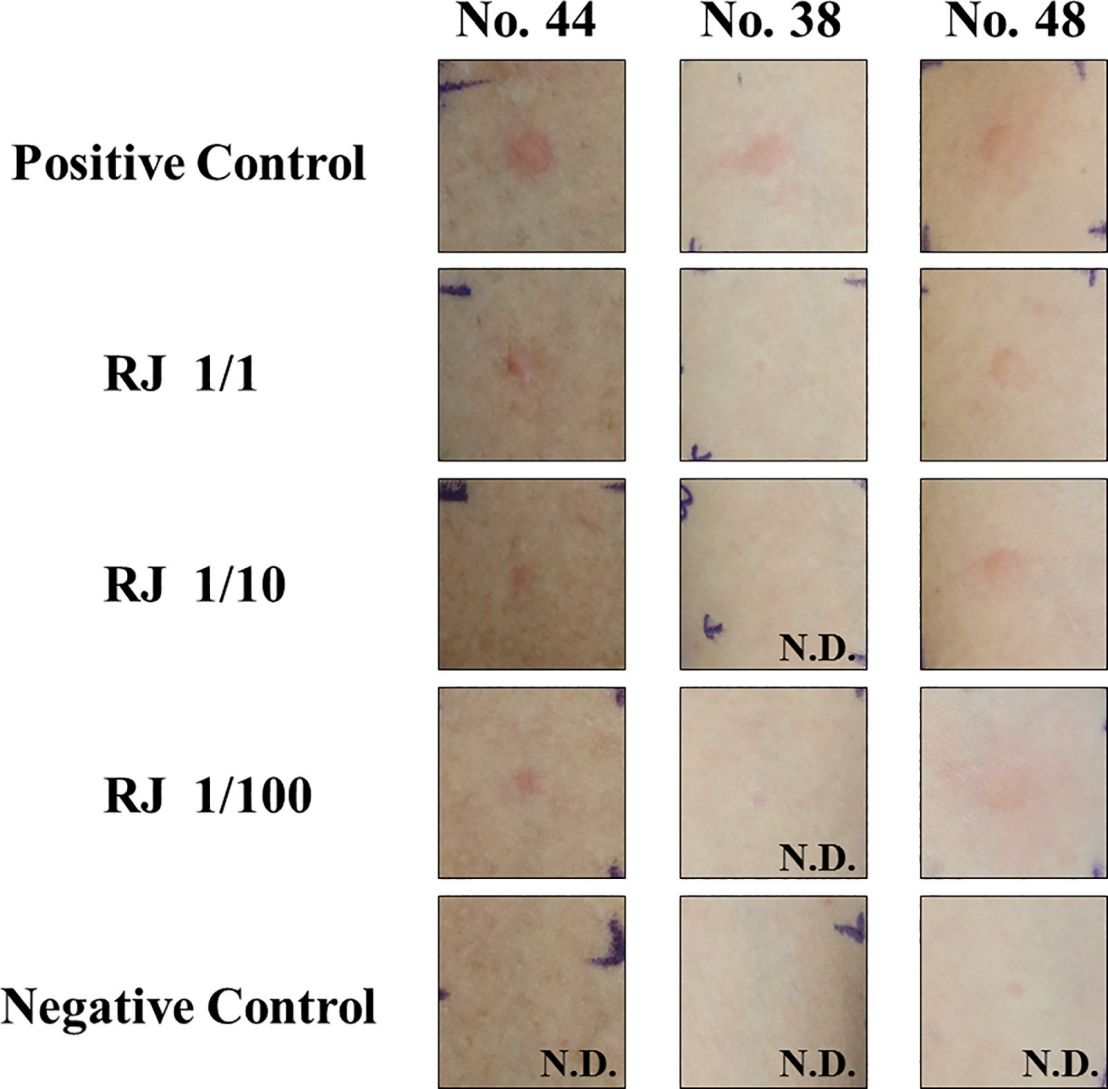
Skin-prick tests for RJ sensitivity in AD patients with no history of RJ exposure. Skin-prick tests were performed in AD patients who participated in this study (subject no. 44, no. 38, and no. 48) with the indicated concentrations of an RJ solution. Subject no. 44 and no. 48 showed positive reactions at all the concentrations tested, and subject no. 38 had a positive reaction at undiluted RJ. A histamine solution was used as a positive control, and a 0.9% saline solution was used as a negative control. N.D. means not detected.

We performed SPTs with various concentrations of an RJ solution in 3 AD patients (no. 44, no. 38, and no. 48). Positive responses were observed in all the subjects tested (Fig 4): subjects no. 44 and no. 48 were positive with undiluted, 1/10, and 1/100 RJ solutions, and No. 38 was positive only with the undiluted RJ solution.

## Discussion

RJ allergy is an exclusive adverse reaction caused by RJ. In the case of RJ, allergic reactions sometimes occur after the first ingestion of an RJ product, and therefore, allergens cross-reactive with RJ have been predicted to exist. In the present study, we found that some AD patients who had never consumed RJ had RJ-binding IgE antibodies. We further demonstrated that RJ cross-reacted with allergen extracts from European HDM, American HDM, snow crab, edible crab, German cockroach, and honeybee venom. To our knowledge, this is the first study to investigate the cross-reactivity between RJ components and a wide range of typical allergens in allergic patients.

Allergy sufferers probably have a higher risk of experiencing allergic reactions to RJ, which can be explained by cross-reactivity. In this study, 33% of AD patients (10 of 30) showed a positive response to RJ in an ELISA experiment. Moreover, significant correlations were observed between the anti-RJ antibody titer and total serum IgE levels and between the anti-RJ antibody titer and EASI scores. The total IgE titer and EASI score are indicators of allergic diathesis and AD severity, respectively. Furthermore, SPTs showed positive results in representative patients. These findings suggest that allergy sufferers should be cautioned about the potential of developing allergic reactions by consuming RJ. These results were consistent with those of previous studies in Australia and Hong Kong, which showed high frequencies of positive results for RJ-binding IgE antibodies in allergic patients [14, 22]. In addition, it was unlikely that the AD patients we studied had been directly sensitized by RJ because they had no previous contact with RJ on the basis of self-reported information. Even though there is a possibility of dual sensitivity, our *in vitro* data suggest that at least some AD patients have cross-reactive antibodies against RJ.

The causal cross-reactive allergen for RJ sensitization was not a single allergen. We confirmed that six out of fourteen typical allergens were promising allergen candidates for cross-reactivity with RJ. The serum from one patient (subject no. 39) exhibited antigenic cross-reactivity between RJ and several allergens, including two mite species, two crab species, a cockroach species, and honeybee venom. Therefore, the RJ-binding IgE antibodies in AD patient serum are thought to be a complex mixture of IgE antibodies with various original specificities. We speculate that HDM allergens largely contribute to the cross-reactivity against RJ because preincubation with HDM allergens drastically decreased the reaction to RJ in an ELISA-inhibition assay. HDM allergens are one of the major inhaled allergens, and serum levels of HDM-specific IgE antibodies are increased in the majority of AD patients [33, 34]. This fact may support our speculation. However, two exceptions were observed. The patients (subject no. 33 and no. 50) who had a high antibody score against European HDM did not show positive reactions to RJ (Table 1 and S2 Table), suggesting possible differences in antigenic epitopes. Although both HDM antigens were prepared from *Dermatophagoides pteronyssinus*, to avoid false positives produced by cross-reactivity, the antigen used in the multiple-allergen simultaneous test had to be more highly purified and processed than the commercially available antigen used in the ELISA-inhibition assay. Thus, the result of the serological test used to detect antibodies against an HDM may not reflect all the specific cross-reactivity to RJ.

Honeybee venom also showed positive cross-reactivity, which can be explained by high homology with the major royal jelly protein family (MRJPs). Approximately 90% of RJ proteins are members of the MRJPs, and MRJP1, MRJP2, and MRJP3 were identified as allergen components of RJ in previous studies [20, 35, 36]. These MRJPs members share high homology, and the identity of the amino acid sequences ranges from 47% to 74% [37]. Honeybee venom contains both MRJP8 and MRJP9 [38, 39], which are registered as api m 11 in the allergen database approved by the World Health Organization and International Union of Immunological Societies (WHO/IUIS) (http://www.allergen.org/) [40]. Therefore, the cross-reactivity between RJ and bee venom can be explained by the high homology of the MRJP members they include. Nonetheless, in some case reports of allergic symptoms occurring after the first intake of RJ, negative results in serological tests for the detection of antibodies against bee venom have been reported [25, 26, 28–30]. Therefore, although honeybee venom is thought to be one of the allergens with the highest cross-reactivity with RJ, it is not the sole cause of allergic symptoms.

The common epitope components shared between RJ and the other allergens are still unclear. One of the possible components could be tropomyosin, a major panallergen in arthropods [41–43]. However, RJ does not contain tropomyosin [35, 44]. To address this issue, we measured concentrations of IgE antibodies specific for Der p 1, Der p 2, Der p 10 (tropomyosin), and Der p 23 in serum samples from RJ-positive/mite-positive patients and RJ-negative/mite-positive patients to examine the common epitope component in the European HDM. No clear correlations between the anti-RJ antibody titer and the concentrations of those specific IgE antibodies were observed (S4 Table). Accordingly, tropomyosin is unlikely to be a common allergen component.

It is speculated that there must be unknown common protein epitopes; therefore, we subsequently conducted protein-BLAST searches (https://blast.ncbi.nlm.nih.gov/) using the MRJP1 amino acid sequence to identify epitope components. However, the BLAST search analysis showed no significant similarity among the following organisms: *Dermatophagoides pteronyssinus*, *Dermatophagoides farinae*, *Pyroglyphidae*, *Blattell*a *germanica*, *Chionoecetes*, and *Cancer pagurus* (search date: October 31, 2019; see S5 Table). These results may reflect the limited information for arthropods in the database.

The allergen extracts from cat dander, Japanese cider pollen, Japanese mugwort pollen, timothy grass pollen, peanut, buckwheat, Alaskan pink shrimp, and silkworm moth did not show cross-reactivity with RJ in this study, suggesting that botanical-origin allergens may have no cross-reactivity with the RJ protein. Vila et al. also used timothy grass pollen and European mugwort pollen as negative controls in an immunoblot-inhibition assay [24]. The Alaskan pink shrimp and silkworm moth are classified as arthropods, and significant correlations were found between the anti-RJ antibody titer and both positive allergens, shrimp and moth, in the ViewAllergy^TM^39 allergen test. Possible reasons for these discrepancies are as follows: the shrimp allergen used in the ViewAllergy^TM^39 test is extracted from not only the Alaskan pink shrimp but also three other species according to the test specifications, and the moth allergen used in our inhibition assay was extracted from only the wing region of the body, which may have limited the proteins included in the extract.

In conclusion, the present study suggest that RJ causes allergic symptoms in allergy sufferers via its cross-reactivity with causative allergens, especially mite and arthropod allergens, although the common epitopes remain unclear. Therefore, people with a history of allergic disease such as AD, asthma, or allergic rhinitis should be cautioned when taking RJ products to allow the safe and successful use of RJ supplements. Further challenge tests are needed to clarify whether allergic patients who are sensitized to mites or arthropods develop allergic symptoms after their first exposure to RJ.

## Supporting information

S1 Table Clinical information and experimental data for RJ-exposed factory workers. (DOCX)

S2 Table ViewAllergy^TM^ 39 classification scores of AD patients. (DOCX)

S3 Table Correlations between royal jelly-specific antibody titers and several blood parameters in AD patients. (DOCX)

S4 Table Allergen component test for *Dermatophagoides pteronyssinus*. (DOCX)

S5 Table *In silico* search for a protein similar to MRJP1. (DOCX)

S1 Raw Images (PDF)
