# Supplementary material for: Studies of royal jelly and associated cross-reactive allergens in atopic dermatitis patients
Source: PLoS One. 2020 Jun 2;15(6):e0233707. doi: 10.1371/journal.pone.0233707 (PMC7266330; doi:10.1371/journal.pone.0233707)
Supplement: S1 Table — (DOCX) [file pone.0233707.s001.docx]

**S1 Table. Clinical information and experimental data for RJ-exposed factory workers.**

| Subject no. | Age | Sex | Onset | History of allergy | Nonspecific IgE (IU/mL) | RJ-specific antibody titer  (fold) | Log_2_ RJ  antibody titer |
| --- | --- | --- | --- | --- | --- | --- | --- |
| 8 | not disclosed | not disclosed | 39 | none | 138 | 2048 | 11 |
| 11 | not disclosed | not disclosed | 35 | egg? | 180 | 256 | 8 |
| 14 | not disclosed | not disclosed | 36 | none | 617 | 1024 | 10 |
