# Supplementary material for: Studies of royal jelly and associated cross-reactive allergens in atopic dermatitis patients
Source: PLoS One. 2020 Jun 2;15(6):e0233707. doi: 10.1371/journal.pone.0233707 (PMC7266330; doi:10.1371/journal.pone.0233707)
Supplement: S2 Table — (DOCX) [file pone.0233707.s002.docx]

**S2 Table. ViewAllergy^TM^ 39 classification scores of AD patients.**

| Subject no. | | 31 | 32 | 33 | 34 | 35 | 36 | 37 | 38 | 39 | 40 | 41 | 42 | 43 | 44 | 45 | 46 | 47 | 48 | 49 | 50 | 51 | 52 | 53 | 54 | 55 | 56 | 57 | 58 | 59 | 60 |
| --- | --- | --- | --- | --- | --- | --- | --- | --- | --- | --- | --- | --- | --- | --- | --- | --- | --- | --- | --- | --- | --- | --- | --- | --- | --- | --- | --- | --- | --- | --- | --- |
| View Allergy^TM^ 39 Class | Orchard grass | 4 | 0 | 1 | 3 | 0 | 0 | 5 | 2 | 3 | 1 | 3 | 1 | 1 | 2 | 0 | 0 | 0 | 3 | 2 | 2 | 4 | 0 | 2 | 2 | 0 | 0 | 3 | 0 | 5 | 0 |
|  | Timothy grass | 4 | 0 | 1 | 3 | 0 | 1 | 5 | 2 | 2 | 1 | 3 | 0 | 1 | 2 | 0 | 0 | 0 | 3 | 2 | 2 | 3 | 0 | 2 | 2 | 0 | 0 | 3 | 0 | 6 | 0 |
|  | Common ragweed | 0 | 0 | 1 | 4 | 1 | 2 | 3 | 2 | 2 | 0 | 2 | 1 | 2 | 2 | 0 | 0 | 0 | 3 | 2 | 2 | 3 | 0 | 2 | 2 | 0 | 0 | 1 | 0 | 4 | 0 |
|  | Mugwort | 1 | 2 | 0 | 3 | 0 | 1 | 3 | 2 | 2 | 0 | 1 | 1 | 0 | 0 | 0 | 1 | 0 | 2 | 2 | 2 | 2 | 0 | 2 | 1 | 0 | 0 | 0 | 0 | 3 | 0 |
|  | Japanese cedar | 3 | 3 | 5 | 5 | 5 | 4 | 5 | 4 | 4 | 3 | 2 | 2 | 5 | 5 | 4 | 4 | 2 | 4 | 4 | 4 | 3 | 1 | 5 | 3 | 1 | 4 | 3 | 2 | 6 | 3 |
|  | Japanese cypress | 2 | 2 | 3 | 4 | 4 | 2 | 4 | 3 | 3 | 2 | 1 | 2 | 3 | 4 | 3 | 3 | 0 | 3 | 2 | 3 | 3 | 1 | 3 | 1 | 1 | 3 | 2 | 0 | 4 | 3 |
|  | Grey alder | 0 | 3 | 4 | 6 | 0 | 0 | 5 | 1 | 2 | 0 | 1 | 0 | 1 | 1 | 0 | 0 | 0 | 3 | 2 | 3 | 3 | 0 | 3 | 0 | 0 | 0 | 0 | 0 | 6 | 0 |
|  | Common silver birch | 0 | 3 | 5 | 6 | 0 | 0 | 5 | 2 | 2 | 0 | 1 | 0 | 0 | 2 | 0 | 0 | 0 | 3 | 1 | 3 | 3 | 0 | 2 | 0 | 0 | 0 | 0 | 0 | 6 | 0 |
|  | House dust mite | 0 | 0 | 6 | 5 | 1 | 6 | 6 | 2 | 6 | 2 | 4 | 4 | 4 | 6 | 4 | 4 | 2 | 5 | 0 | 6 | 5 | 0 | 3 | 4 | 1 | 2 | 3 | 2 | 6 | 3 |
|  | House dust 1 | 0 | 0 | 6 | 4 | 3 | 6 | 6 | 2 | 5 | 2 | 4 | 3 | 4 | 5 | 3 | 4 | 1 | 5 | 2 | 6 | 5 | 0 | 3 | 4 | 1 | 2 | 3 | 2 | 5 | 2 |
|  | *Candida albicans* | 0 | 1 | 3 | 3 | 2 | 3 | 3 | 4 | 3 | 0 | 2 | 3 | 0 | 2 | 0 | 2 | 0 | 4 | 0 | 4 | 2 | 1 | 0 | 3 | 1 | 2 | 3 | 2 | 2 | 0 |
|  | *Alternaria alternata* | 0 | 0 | 2 | 2 | 1 | 2 | 2 | 4 | 2 | 0 | 0 | 1 | 0 | 0 | 0 | 2 | 0 | 3 | 0 | 3 | 1 | 0 | 0 | 2 | 0 | 2 | 2 | 1 | 1 | 0 |
|  | *Aspergillus fumigatus* | 0 | 0 | 3 | 2 | 1 | 2 | 3 | 3 | 2 | 0 | 0 | 3 | 0 | 1 | 0 | 1 | 0 | 3 | 0 | 3 | 2 | 0 | 2 | 2 | 1 | 1 | 2 | 2 | 2 | 0 |
|  | *Malassezia spp.* | 2 | 2 | 4 | 4 | 4 | 5 | 5 | 4 | 4 | 0 | 3 | 4 | 2 | 4 | 0 | 3 | 0 | 5 | 0 | 4 | 4 | 2 | 2 | 5 | 2 | 3 | 3 | 4 | 2 | 0 |
|  | Cat dander | 0 | 1 | 2 | 1 | 2 | 4 | 3 | 2 | 3 | 0 | 4 | 0 | 0 | 4 | 0 | 2 | 0 | 3 | 2 | 0 | 3 | 0 | 3 | 0 | 0 | 0 | 0 | 0 | 4 | 0 |
|  | Dog dander | 0 | 1 | 3 | 2 | 3 | 3 | 4 | 2 | 3 | 0 | 5 | 1 | 2 | 2 | 0 | 2 | 0 | 3 | 2 | 2 | 2 | 0 | 1 | 1 | 0 | 0 | 2 | 1 | 3 | 1 |
|  | Wheat | 0 | 0 | 1 | 3 | 1 | 1 | 2 | 2 | 2 | 0 | 2 | 1 | 1 | 1 | 0 | 1 | 0 | 2 | 4 | 1 | 2 | 0 | 0 | 1 | 1 | 1 | 1 | 0 | 3 | 0 |
|  | Soybean | 0 | 0 | 0 | 3 | 0 | 0 | 2 | 2 | 2 | 0 | 0 | 0 | 0 | 1 | 0 | 0 | 0 | 1 | 2 | 1 | 0 | 0 | 0 | 0 | 0 | 0 | 0 | 0 | 3 | 0 |
|  | Rice | 0 | 0 | 1 | 3 | 0 | 0 | 2 | 2 | 2 | 0 | 3 | 0 | 0 | 1 | 0 | 0 | 0 | 2 | 2 | 1 | 1 | 0 | 2 | 0 | 0 | 0 | 0 | 0 | 3 | 0 |
|  | Sesame seed | 0 | 0 | 3 | 3 | 1 | 2 | 3 | 2 | 3 | 0 | 1 | 2 | 1 | 2 | 0 | 0 | 0 | 2 | 2 | 2 | 2 | 0 | 0 | 1 | 0 | 0 | 2 | 1 | 3 | 0 |
|  | Tuna (Yellowfin) | 0 | 0 | 0 | 0 | 0 | 0 | 0 | 1 | 2 | 0 | 0 | 0 | 0 | 0 | 0 | 0 | 0 | 0 | 0 | 2 | 0 | 0 | 0 | 0 | 0 | 0 | 0 | 0 | 1 | 0 |
|  | Salmon (Atlantic) | 0 | 0 | 0 | 0 | 0 | 0 | 1 | 1 | 2 | 0 | 0 | 0 | 0 | 0 | 0 | 0 | 0 | 1 | 0 | 2 | 0 | 0 | 0 | 0 | 0 | 0 | 0 | 0 | 1 | 0 |
|  | Chub mackerel | 0 | 0 | 0 | 1 | 0 | 0 | 0 | 0 | 0 | 0 | 0 | 0 | 0 | 0 | 0 | 0 | 0 | 0 | 0 | 0 | 0 | 0 | 0 | 0 | 0 | 0 | 0 | 0 | 1 | 0 |
|  | Shrimp | 0 | 0 | 1 | 3 | 1 | 2 | 1 | 2 | 2 | 0 | 0 | 1 | 1 | 3 | 0 | 0 | 0 | 2 | 1 | 2 | 1 | 0 | 0 | 2 | 2 | 2 | 0 | 1 | 2 | 0 |
|  | Crab | 0 | 0 | 0 | 0 | 0 | 2 | 1 | 1 | 3 | 0 | 0 | 0 | 0 | 3 | 0 | 0 | 0 | 2 | 1 | 0 | 0 | 0 | 0 | 1 | 0 | 0 | 0 | 0 | 2 | 0 |
|  | Cow's milk | 0 | 0 | 0 | 0 | 0 | 0 | 0 | 0 | 0 | 0 | 0 | 0 | 0 | 1 | 0 | 0 | 0 | 0 | 3 | 1 | 0 | 0 | 0 | 0 | 0 | 0 | 0 | 0 | 1 | 0 |
|  | Beef | 0 | 0 | 0 | 1 | 0 | 1 | 1 | 1 | 1 | 0 | 0 | 1 | 0 | 0 | 0 | 0 | 0 | 1 | 4 | 3 | 1 | 0 | 0 | 1 | 1 | 0 | 0 | 0 | 1 | 0 |
|  | Chicken | 0 | 0 | 0 | 0 | 0 | 0 | 0 | 0 | 1 | 0 | 0 | 0 | 0 | 1 | 0 | 0 | 0 | 2 | 0 | 0 | 0 | 0 | 0 | 0 | 0 | 0 | 0 | 0 | 1 | 0 |
|  | Pork | 0 | 0 | 1 | 1 | 1 | 1 | 2 | 0 | 0 | 0 | 0 | 1 | 0 | 0 | 1 | 0 | 0 | 0 | 3 | 2 | 1 | 0 | 0 | 0 | 0 | 0 | 0 | 0 | 1 | 0 |
|  | Egg white | 0 | 0 | 0 | 0 | 0 | 0 | 0 | 0 | 2 | 0 | 2 | 0 | 0 | 0 | 0 | 0 | 0 | 0 | 2 | 0 | 0 | 0 | 0 | 0 | 0 | 0 | 0 | 0 | 1 | 0 |
|  | Ovomucoid | 0 | 0 | 0 | 0 | 0 | 0 | 0 | 0 | 2 | 0 | 0 | 0 | 0 | 0 | 0 | 0 | 0 | 0 | 0 | 0 | 0 | 0 | 0 | 0 | 0 | 0 | 0 | 0 | 1 | 0 |
|  | Buckwheat | 0 | 0 | 0 | 3 | 0 | 1 | 2 | 2 | 2 | 0 | 1 | 1 | 2 | 1 | 0 | 0 | 0 | 1 | 2 | 1 | 1 | 0 | 0 | 1 | 0 | 0 | 0 | 0 | 3 | 0 |
|  | Peanut | 0 | 0 | 0 | 3 | 0 | 0 | 3 | 1 | 2 | 0 | 0 | 0 | 0 | 1 | 0 | 0 | 0 | 1 | 2 | 2 | 1 | 0 | 1 | 0 | 2 | 0 | 0 | 0 | 3 | 0 |
|  | Apple | 0 | 2 | 3 | 4 | 1 | 1 | 4 | 2 | 2 | 0 | 1 | 1 | 1 | 2 | 0 | 0 | 0 | 2 | 2 | 2 | 1 | 0 | 2 | 1 | 0 | 0 | 0 | 0 | 4 | 0 |
|  | Kiwi | 0 | 0 | 1 | 3 | 0 | 1 | 3 | 1 | 2 | 0 | 1 | 1 | 0 | 1 | 0 | 0 | 0 | 1 | 2 | 1 | 2 | 0 | 1 | 0 | 0 | 0 | 2 | 0 | 3 | 0 |
|  | Banana | 0 | 0 | 0 | 4 | 1 | 1 | 2 | 2 | 2 | 0 | 1 | 1 | 1 | 2 | 0 | 0 | 0 | 2 | 2 | 1 | 1 | 0 | 1 | 1 | 1 | 1 | 1 | 1 | 3 | 0 |
|  | Latex | 0 | 0 | 0 | 3 | 0 | 0 | 3 | 1 | 1 | 0 | 3 | 0 | 1 | 1 | 0 | 0 | 0 | 1 | 2 | 1 | 1 | 0 | 1 | 0 | 0 | 0 | 0 | 0 | 3 | 0 |
|  | Cockroach (German) | 0 | 0 | 1 | 4 | 1 | 2 | 2 | 1 | 2 | 0 | 0 | 1 | 1 | 3 | 0 | 0 | 0 | 2 | 3 | 2 | 1 | 0 | 0 | 2 | 1 | 1 | 0 | 1 | 2 | 0 |
|  | Moth | 0 | 1 | 1 | 2 | 1 | 2 | 2 | 2 | 2 | 0 | 0 | 2 | 1 | 3 | 0 | 2 | 0 | 2 | 2 | 3 | 1 | 0 | 1 | 4 | 2 | 2 | 2 | 1 | 2 | 0 |
