# Supplementary material for: Studies of royal jelly and associated cross-reactive allergens in atopic dermatitis patients
Source: PLoS One. 2020 Jun 2;15(6):e0233707. doi: 10.1371/journal.pone.0233707 (PMC7266330; doi:10.1371/journal.pone.0233707)
Supplement: S3 Table — (DOCX) [file pone.0233707.s003.docx]

**S3 Table. Correlations between royal jelly-specific antibody titers and several blood parameters in AD patients.**

| Subject no. | RJ-specific antibody titer  (fold) | TARC  (pg/mL) | Eosinophils (%) | Eosinophils (cells/μL) | LD- (124-222 U/L) |
| --- | --- | --- | --- | --- | --- |
| 31 | 0 | 268 | 2.4 | 133 | N/A |
| 32 | 0 | 14439 | 31.7 | 2447 | 194 |
| 33 | 0 | 6167 | 18.6 | 1793 | 201 |
| 34 | 32 | 1202 | 4.0 | 221 | N/A |
| 35 | 0 | 1272 | 5.7 | 536 | 306 |
| 36 | 512 | 854 | 3.9 | 381 | N/A |
| 37 | 128 | 3329 | 32.5 | 2379 | 409 |
| 38 | 8 | 2030 | 12.5 | 1325 | 218 |
| 39 | 2048 | 4937 | 13.4 | 829 | 382 |
| 40 | 0 | 539 | 2.6 | 287 | N/A |
| 41 | 0 | 759 | 7.0 | 859 | 233 |
| 42 | 4 | 770 | 4.4 | 272 | 196 |
| 43 | 0 | 260 | 5.3 | 359 | N/A |
| 44 | 1024 | 4419 | 9.4 | 619 | 319 |
| 45 | 0 | 1016 | 2.6 | 217 | N/A |
| 46 | 0 | 266 | 3.9 | 190 | 125 |
| 47 | 0 | 282 | 0.0 | 0 | 234 |
| 48 | 64 | 1002 | 15.0 | 1067 | 195 |
| 49 | 0 | 1180 | N/A | N/A | 222 |
| 50 | 0 | 3565 | 8.0 | 758 | 422 |
| 51 | 16 | 1932 | 8.9 | 473 | N/A |
| 52 | 0 | 4758 | 9.0 | 883 | 143 |
| 53 | 0 | 451 | 7.3 | 418 | N/A |
| 54 | 0 | 26845 | 26.0 | 2192 | 206 |
| 55 | 0 | 4752 | 10.5 | 549 | 253 |
| 56 | 0 | 11151 | 7.0 | 924 | 402 |
| 57 | 0 | 333 | 5.1 | 229 | 150 |
| 58 | 8 | 525 | 3.0 | 72 | N/A |
| 59 | 0 | 938 | 9 | 498 | N/A |
| 60 | 0 | 458 | 2.5 | 208 | 176 |
| Spearman's rank correlation coefficient for the anti-RJ antibody titer | - | 0.178 | 0.244 | 0.129 | 0.292 |
| *p* value | - | 0.347 | 0.203 | 0.506 | 0.211 |
