# Supplementary material for: Studies of royal jelly and associated cross-reactive allergens in atopic dermatitis patients
Source: PLoS One. 2020 Jun 2;15(6):e0233707. doi: 10.1371/journal.pone.0233707 (PMC7266330; doi:10.1371/journal.pone.0233707)
Supplement: S4 Table — (DOCX) [file pone.0233707.s004.docx]

**S4 Table. Allergen component test for *Dermatophagoides pteronyssinus.***

| Subject no. | Allergen component | | | | Log2 RJ antibody titer | House dust mite class |
| --- | --- | --- | --- | --- | --- | --- |
|  | Der p 1 | Der p 2 | Der p 10 | Der p 23 |  |  |
| 39 | 68.2 | >100 | 0.4 | 43.2 | 11 | 6 |
| 44 | 42.7 | 70 | 15.9 | 86.1 | 10 | 6 |
| 36 | >100 | >100 | 0.229 | >100 | 9 | 6 |
| 33 | 54.7 | >100 | <0.1 | 12.8 | 0 | 6 |
| 50 | 89.6 | >100 | 0.283 | 54.7 | 0 | 6 |
