# Supplementary material for: Studies of royal jelly and associated cross-reactive allergens in atopic dermatitis patients
Source: PLoS One. 2020 Jun 2;15(6):e0233707. doi: 10.1371/journal.pone.0233707 (PMC7266330; doi:10.1371/journal.pone.0233707)
Supplement: S5 Table — (DOCX) [file pone.0233707.s005.docx]

**S5 Table. *In silico* search for a protein similar to MRJP1.**

| Query | | Search set/ subject | Program | Result |
| --- | --- | --- | --- | --- |
| Name | Accession no. |  |  |  |
| MRJP1 | AF000633 | Dermatophagoides pteronyssinus (taxid:6956) | Standard Nucleotide BLAST Database: Nucleotide collection (nr/nt) Highly similar sequences (megablast)  Search date: October 31, 2019 https://blast.ncbi.nlm.nih.gov/ | No significant similarity found. |
|  |  | Dermatophagoides farinae (taxid:6954) |  | No significant similarity found. |
|  |  | Dermatophagoides (taxid:6953) |  | No significant similarity found. |
|  |  | Dermatophagoidinae (taxid:474036) |  | No significant similarity found. |
|  |  | Pyroglyphidae (taxid:6952) |  | No significant similarity found. |
|  |  | Unclassified Pyroglyphidae (taxid:1663131) |  | No significant similarity found. |
|  |  | Blattella germanica (taxid:6973) |  | No significant similarity found. |
|  |  | Blattella (taxid:6972) |  | No significant similarity found. |
|  |  | Chionoecetes (taxid:41209) |  | No significant similarity found. |
|  |  | Chionoecetes japonicus (taxid:290538) |  | No significant similarity found. |
|  |  | Cancer pagurus (taxid:6755) |  | No significant similarity found. |
