# Supplementary material for: Studies of royal jelly and associated cross-reactive allergens in atopic dermatitis patients
Source: PLoS One. 2020 Jun 2;15(6):e0233707. doi: 10.1371/journal.pone.0233707 (PMC7266330; doi:10.1371/journal.pone.0233707)

Raw images _ Fig.1 Western blotting

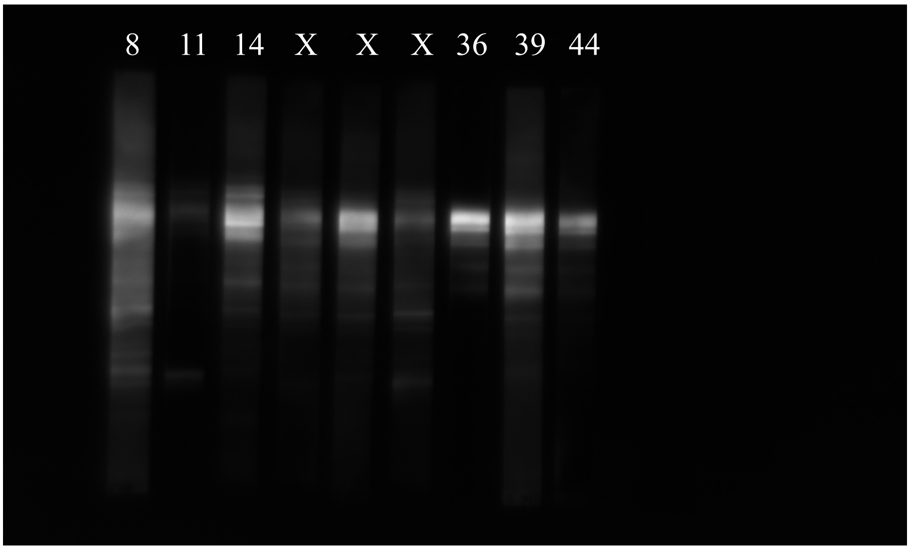

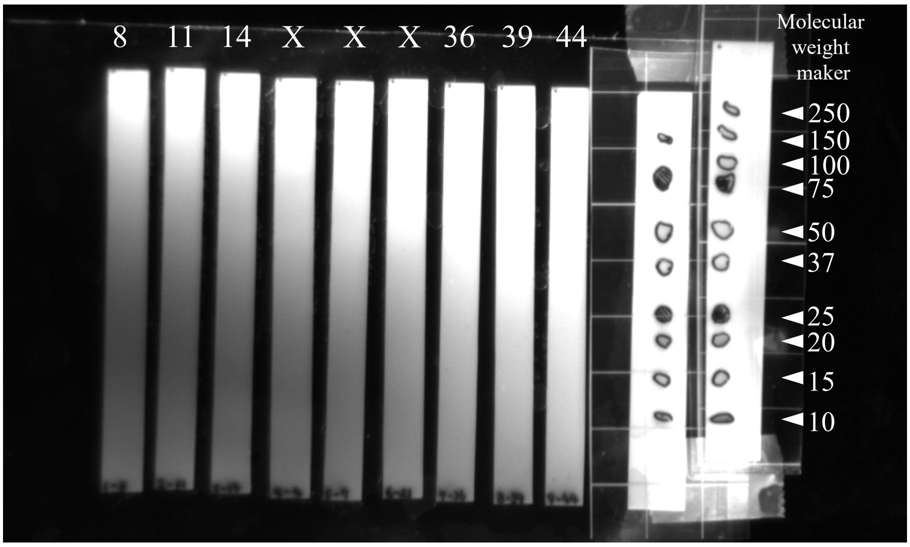

RJ factory worker AD patient

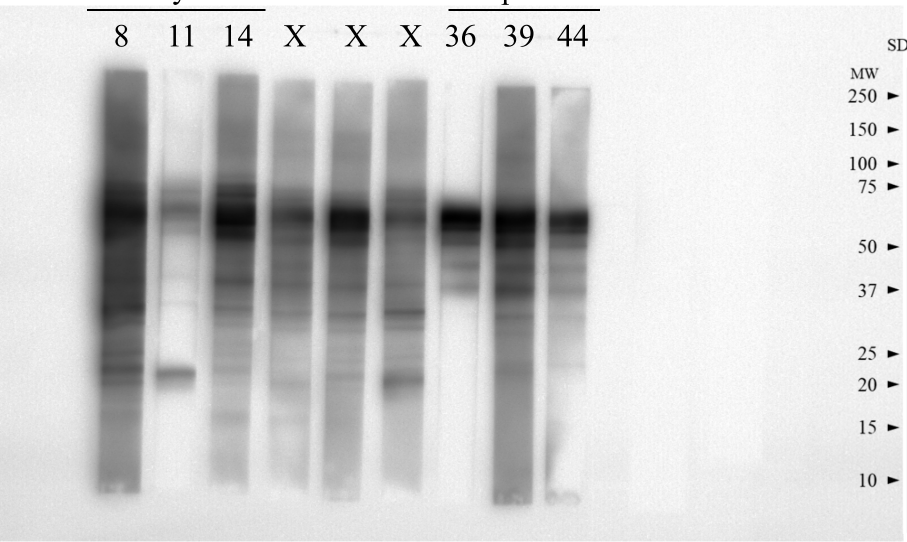

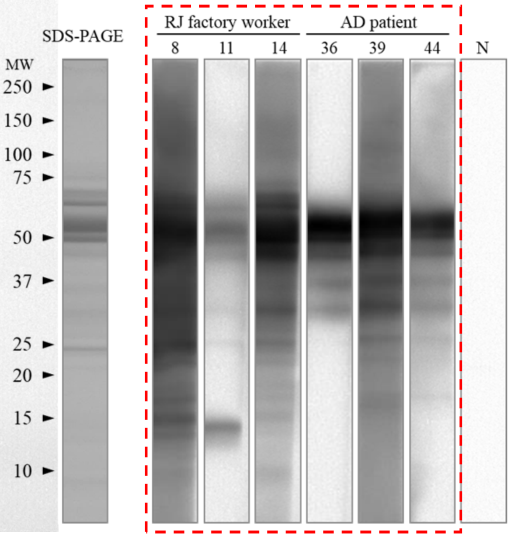

Raw images _ Fig.1 SDS-PAGE

MW

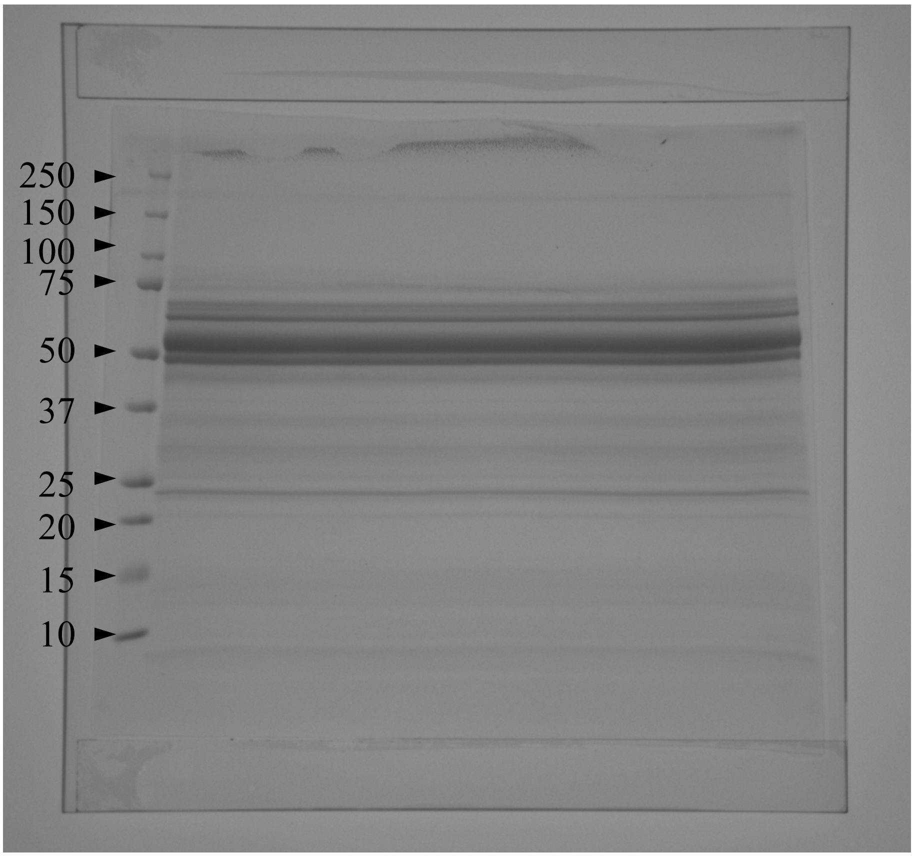

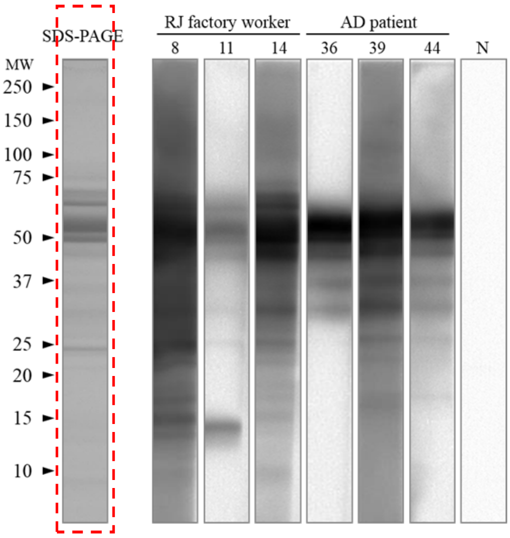

Raw images _ Fig.1 Western blotting (Negative control)

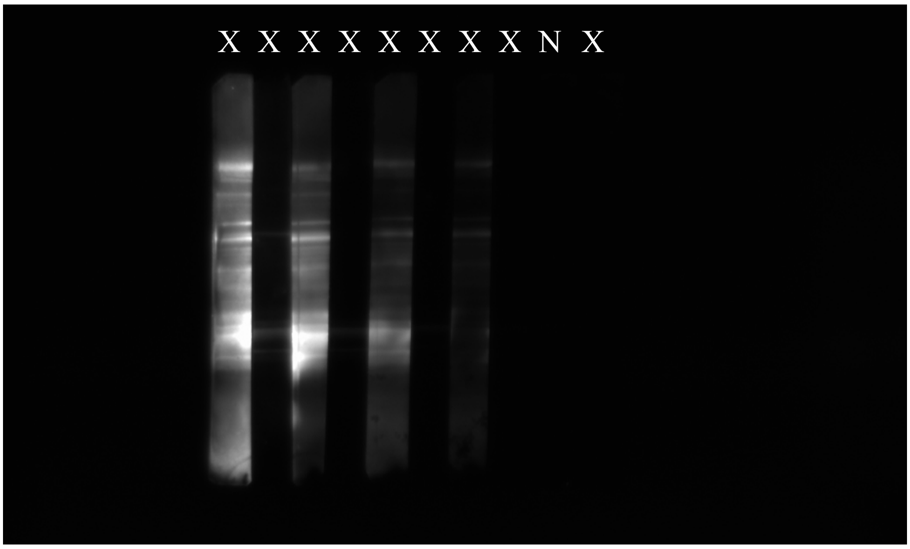

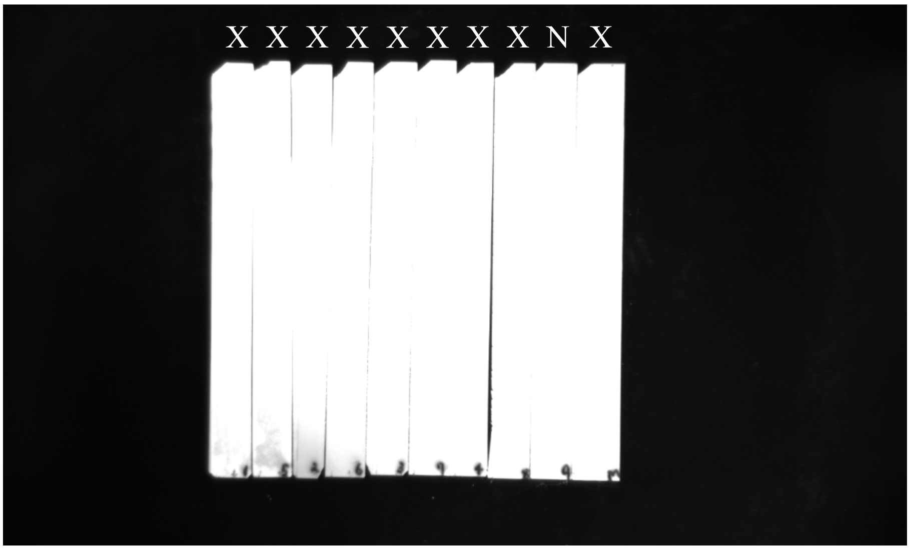

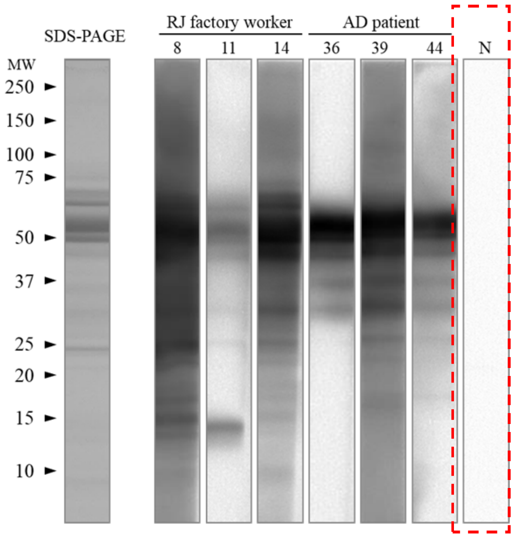

Supplement: S1 Raw Images — (PDF) [file pone.0233707.s006.pdf]
